# Polish Pharmacy Students’ Readiness, Qualifications, Competences, Relevance, Motivation and Effectiveness to Promote Health in Community Pharmacies

**DOI:** 10.3390/ijerph182413227

**Published:** 2021-12-15

**Authors:** Dorota Raczkiewicz, Bartosz Kobuszewski, Beata Sarecka-Hujar, Adrianna Pawełczak-Barszczowska, Iwona Bojar

**Affiliations:** 1 Department of Medical Statistics, School of Public Health, Centre of Postgraduate Medical Education, Kleczewska str 61/63, 01-826 Warsaw, Poland; dorota.bartosinska@gmail.com; 2 Department of Medical Law and Decisions, School of Public Health, Centre of Postgraduate Medical Education, Kleczewska str 61/63, 01-826 Warsaw, Poland; 3 Department of Basic Biomedical Science, Faculty of Pharmaceutical Sciences in Sosnowiec, Medical University of Silesia in Katowice, Kasztanowa str 3, 41-200 Sosnowiec, Poland; bsarecka-hujar@sum.edu.pl; 4 Zentiva, Bonifraterska str 17, 00-203 Warsaw, Poland; apawelczak81@gmail.com; 5 Department of Women’s Health, Institute of Rural Health, Jaczewskiego str 2, 20-090 Lublin, Poland; iwonabojar75@gmail.com

**Keywords:** pharmacy students, pharmacy, pharmacists, health promotion, health education, pharmaceutical care, implementation of pharmaceutical care within the health system

## Abstract

Background: One of the parts of the broadly understood pharmaceutical care is health promotion. Therefore, the study aimed to find out how pharmacy students in Poland assess their own readiness to promote health in pharmacies and their own qualifications, competences, relevance, motivation and effectiveness of health promotion in pharmacies. Methods: The study conducted in 2019 comprised 206 pharmacy students from Poland. The authors’ survey questionnaire had two parts: Readiness to promote health in pharmacies; and Qualifications, competences, relevance, motivation and effectiveness of health promotion in pharmacies. Results: The students assessed the system solutions regarding health promotion as insufficient. The highest assessment was given to their own readiness to promote health. In between those was assessment of readiness to promote health by pharmacists as an occupational group. Readiness to promote health at a workplace in a pharmacy was assessed higher than in a local community. The students gave the highest assessments to the relevance and motivation to promote health, and the lowest to their own competences to promote health. In between those, their qualifications and effectiveness to health promotion in pharmacies. were assessed. Conclusions: Pharmacy students consider themselves ready and motivated to promote health, that is of a great importance in their opinion, and they could potentially play an important role in improving the health care of patients.

## 1. Introduction

In the 1970s, Bush and Johnson [1] pointed out that too few pharmacists are involved in public health activities and that their potential in public health is not recognized during their education. According to the authors, there is a need for pharmacy graduates to be able to participate in public health at both a micro-level (in relation to an individual patient) as well as a macro-level (focus on the health condition of the community) [1]. Nowadays, both actions within an individual patient’s health condition as well as the whole community’s health condition are commonly practiced by pharmacists in a growing number of countries [2].

The scope of pharmaceutical care includes the following activities: rationalization of pharmacotherapy, individual conversation/contact with the patient enabling full knowledge on medical problems, health education, as well as a cooperation with the general physician consisting in monitoring side effects and drug interactions. Medical universities, educating pharmacy students as well as pharmacists in the field of postgraduate training, play a huge role in the proper development and functioning of pharmaceutical care. Currently, pharmaceutical studies in Poland last 5 years, after which it is obligatory to complete a 6-month professional practice in a pharmacy. After the graduation, students obtain a master’s degree in pharmacy. 

There is a nationwide Polish graduate tracking system monitoring the economic fate of university graduates, but it does not contain data on the current workplace. However, some of the universities were gathering this information. According to the data of the Medical University of Warsaw on the 2017 graduates [3] and the Jagiellonian University in Cracow on the 2014 graduates [4], 30% and 73% of Masters of Pharmacy, respectively, work at community pharmacies. The graduates from Warsaw also work at pharmaceutical companies (38%) and carry out clinical trials (18%), while the graduates from Cracow work at medical universities (11.5%), hospitals (3.8%) and pharmaceutical companies (3.8%). Data from other universities are not available.

In Poland, the rules of practicing the profession of a pharmacist are determined by the Act on the Profession of Pharmacist of 2020 [5]. It defines pharmaceutical care as a health service in the sense of the Act on Health Care Services Financed from Public Funds of 2004 [6], which means that pharmacies having a contract with the National Health Fund will receive additional funds for conducting a pharmaceutical care. The Act states that pharmaceutical care is provided by a pharmacist and constituting a documented process, in which the pharmacist, in cooperation with the patient and the patient’s doctor, and, if necessary, with representatives of other medical professions, ensures a proper course of individual pharmacotherapy, including, among others, developing an individual pharmaceutical care plan, taking into account the patient’s medicines problems. It is done in order to define therapeutic goals that can be achieved by the patient who is using pharmacotherapy and to indicate ways of solving detected drug problems, with particular emphasis on health education, health promotion and healthy lifestyle and health prevention. It also states that professional tasks of a pharmacist include conducting preventive, educational and health promotion activities. 

In order to properly implement these extra activities, a pharmacist needs to possess an extensive knowledge not only in the field of pharmacology and pharmacodynamics, drug formulation technology and physiology, but also a knowledge of the basics of psychology and economics [7]. 

Actions aimed to increase the involvement of pharmacists in pharmaceutical care were observed before the enacting of the new law. Education in the field of pharmaceutical care has become a priority task for pharmaceutical faculties, which should result in the pharmacist being the first advisor on the patient’s health problems. It should in turn reduce unnecessary medical appointments, and thus reduce economic burden. In 2006, the “Practical Pharmacy in community pharmacies” class was included in the fifth-year curriculum of pharmaceutical studies, during which future pharmacists learn pharmaceutical counseling, databases of drugs, drug interactions and other problems with drug usage, pharmaceutical care for chronically ill patients, keeping records and communicating with the patient [6]. Mock pharmacist-patient interactions are played out by students, and this plays an important role in the process of teaching pharmaceutical care to pharmacy students. Such training takes place in the training pharmacies which are located in most Polish medical universities, among others in Gdańsk, Sosnowiec, Kraków, Bydgoszcz and Poznań [7].

Pharmacists, in addition to monitoring and dispensing medicine, are also involved in the treatment of chronic diseases, the treatment of common illnesses and accidents, healthy lifestyle promotion [8,9]. Similarly, at present, pharmacy students are involved, among others, in diabetes prevention, in consultations on vaccination against influenza, in the assessment of drug addiction in patients, in antithrombotic prevention of heart disease, the promotion of organ transplantation, in the education on the H1N1 flu pandemic risk factors, or the activities related to the reduction of nicotine smoking [10,11,12,13,14,15,16]. The literature data also emphasize the need for an improved scientific cooperation between pharmacy students and medical clinics [17]. There are many reports that positively assess the attitude of students and their role in public health [18,19,20]. Abdelhalim et al. [21] indicated that third year pharmacy students have a great potential to recognize various types of medical problems and to suggest appropriate solutions. 

The research conducted so far has focused on the Polish students’ opinions on pharmaceutical care, which also contained health promotion, provided in pharmacies [22] or in the neonatal intensive care units [23]. Studies performed in Canada [24], Qatar [18] and Malaysia [25] showed that pharmacy students find themselves prepared to perform tasks in the area of health promotion. However, these studies wanted to assess the influence of taking part in public health events as a part of educational process on students’ knowledge and skill development.

The aim of the present study was to find out how pharmacy students in Poland assess their own readiness to promote health in pharmacies as well as their own qualifications, competences, relevance, motivation and effectiveness of health promotion in pharmacies. 

## 2. Materials and Methods

### 2.1. Study Group

The study was carried out in April and November 2019 among all the fourth-year pharmacy students (one year before their graduation) in academic years 2018/2019 and 2019/2020, respectively, in the Medical University of Lublin, Poland.

This sample is representative of all medical universities in Poland as they all have the same curriculum, and all pharmacies in all the regions across Poland function in the same manner and are governed by the same legal regulations. 

The students were given the questionnaires before lectures. The participation in the survey was voluntary and anonymous. We handed out 250 questionnaires and received 206 completed questionnaires back. The response rate was 82.5%. 

### 2.2. Survey Questionnaires

The two survey questionnaires which were used were written by the authors. These questionnaires were previously used in the two studies on pharmacy staff [26,27]. The first questionnaire concerned the readiness to promote health in pharmacies, while the second questionnaire concerned qualifications, competences, relevance, motivation and effectiveness of health promotion in pharmacies.

Both questionnaires were piloted on a sample of 30 respondents (20 pharmacy students and 10 pharmacy staff). As a result of the pilot study, some questions have been modified.

### 2.3. The Questionnaire of Readiness to Promote Health in Pharmacies

In order to assess Polish pharmacy students’ readiness to promote health in pharmacies, the Scale of Social Readiness by Gaś has been adopted [28]. Our questionnaire included a total of 32 items in three domains: systemic solutions for health promotion (16 items), readiness of a professional group (10 items) and personal readiness to promote health (6 items) [26]. This questionnaire is included as Supplementary Material S1.

Systemic solutions are related to relevant legal acts, certain activities undertaken by pharmacists, organizations and institutions, cooperation between various professional groups and financial support of health promotion in pharmacies in Poland. The readiness to promote health by pharmacists as a professional group is determined by the following questions: are pharmacists as a professional group prepared to promote health in terms of knowledge and methodology, and whether they have motivation, space, organizational and interpersonal skills. Personal readiness of pharmacists to promote health is demonstrated by the following questions: are they personally ready and committed, and whether they have organizational and interpersonal skills to promote health.

All items were rated on a 1−10 scale where: 1 equaled a ‘definitely no’ and 10 equaled a ‘definitely yes’.

We calculated the ratings of the 3 domains: systemic solutions for health promotion (as a mean of 16 items in this domain), readiness of a professional group (as a mean of 10 items in this domain) and personal readiness to promote health (as a mean of 6 items in this domain).

### 2.4. The Questionnaire of Qualifications, Competences, Relevance, Motivation and Effectiveness of Health Promotion in Pharmacies

This questionnaire is included as Supplementary Material S2. This questionnaire included 5 questions to pharmacy students [27]:

1. How do you assess your qualifications (knowledge and skills) to promote health in a pharmacy in the following items?
2. How do you assess your formal competences (an individual’s capacity to perform job responsibilities) to promote health in a pharmacy in the following items?
3. How do you assess relevance of health promotion in a pharmacy in the following items?
4. How do you assess your motivations to promote health in a pharmacy in the following items?
5. How do you assess your effectiveness in health promotion in a pharmacy in the following items?

Each question contained 36 items in 3 subdomains: health knowledge (11 items); disease prevention (9 items); coping with health problems (16 items). In total, pharmacy students evaluated 180 items (5 questions for 36 items).

All items were assessed by the respondents from 1 to 10 scale; where 1 was ‘very low’, 10 was ‘very high’.

We calculated the ratings of the 5 domains: qualifications (question 1), competences (question 2), relevance (question 3), motivations (question 4) and effectiveness (question 5) as a mean of 36 items in each domain. We also calculated the rating of the 3 subdomains in each domain: health knowledge (as a mean of 11 items); disease prevention (as a mean of 9 items); coping with health problems (as a mean of 16 items).

### 2.5. Statistical Methods

The statistical analysis was conducted with STATISTICA 12 software (Statsoft, Kraków, Poland). An arithmetic mean (M) and a standard deviation (SD) were estimated for every item, every subdomain and every domain. 

Cronbach’s alpha and average inter-correlation coefficient r among the items were used to test reliability and internal coherence for the items within each subdomain and each domain.

The following statistical tests were used:

- one sample *t* test against a value of 5.5 to check whether assessment of a item, a subdomain or a domain of health promotion is neutral or statistically significantly positive or significantly negative;
- two paired samples *t* test to compare readiness to promote health between at a workplace and in a local community.

The scale in both survey questionnaires had a positive direction i.e., the higher the score was, the better the assessment of issues concerning promotion was. The middle of the scale 1–10 was 5.5 so replies statistically significantly lower than 5.5 were considered as negative, replies statistically significantly higher than 5.5 were considered as positive and replies statistically not significantly different from 5.5 were considered as neutral (neither positive nor negative).

The significance level was assumed at 0.05.

## 3. Results

The study consisted of 188 females (91.26%) and 18 males (8.74%).

### 3.1. Pharmacy Students’ Assessment of Readiness to Promote Health in Pharmacies

The scale reliability and internal coherence for all 32 items and for items within the three domains separately: systemic solutions for health promotion, readiness of a professional group and personal readiness to promote health have been tested (Table 1). The total scale of readiness to health promotion had α = 0.948 and r = 0.370. The individual domains had also Cronbach’s α values above 0.9 and strong inter-correlations among the items, which allows 3 domains and the total scale to be used in further analyses.

The surveyed pharmacy students assessed their readiness to promote health in pharmacies negatively (on average 4.9 ± 1.3, significantly below the mid-point on a 1–10 scale (Table 1). System solutions regarding health promotion were assessed significantly the lowest (4.0 ± 1.3 on average). The readiness to promote health by pharmacists as an occupational group was assessed a little higher (5.0 ± 1.6 on average), however both of these assessments were negative. On the other hand, the pharmacy students assessed their own readiness to promote health in pharmacies positively (5.8 ± 1.7 on average, significantly above the scale mid-point).

Among the system solutions regarding health promotion in pharmacies (Table 2), the following questions were assessed the lowest: Does pharmacy professional association take any action aimed at setting a financial framework for health promotion (3.3 on average) as well as a system of financial support for pharmacies to perform health promotion activities, including health education, both at a workplace and in the local communities (3.4 on average). 

The best assessments were given to clear regulations that define and support a role of pharmacists in health promotion, institutions or organizations that provide professional training for pharmacists regarding health promotion, clearly defined concept of health promotion that includes prevention, local health policy and health education.

Regarding the readiness to promote health as an occupational group (Table 3), the pharmacy students gave the highest assessments to the knowledge on health promotion and health education at a workplace. On the other hand, the knowledge on health promotion in the local community was assessed neutrally and significantly lower. What was also assessed neutrally was a positive motivation for health promotion and health education at a workplace and in the local community as well as readiness to promote health and health education at a workplace in terms of methodology (objectives, forms, methods, influencing, methods of diagnosing patient/client, methods of evaluation and other). The knowledge on the methodology of health promotion in the local community, initiation of activities aimed at increasing their readiness to promote health and appropriate conditions (premises, organizational, interpersonal) at the workplace and in local community were all assessed negatively. The knowledge, methodology and conditions to promote health at the workplace were assessed significantly higher than the knowledge, methodology and conditions to promote health in the local community.

Regarding the questions on the students’ personal readiness to promote health (Table 4), the students positively assessed the following aspects: their readiness to recognize and meet health expectations of patients/pharmacy clients to a larger extent than it is necessary for regular buy/sell relations, their readiness to promote health at a workplace and in the local community sufficient for effective actions. Neutral assessments were given to social attitude and personal relationships at a workplace favor health promotion and health education, own effectiveness at health promotion, having appropriate premises and organizational setting for health promotion activities. It is worth pointing out that neither of them was assessed negatively.

### 3.2. Pharmacy Students’ Assessment of Preparation, Competences, Relevance, Motivation and Effectiveness of Health Promotion in Pharmacies

The scale reliability and internal coherence for 5 domains and the 3 subdomains in every 5 domains (a total of 15 subdomains) were tested (Table 5). High values of Cronbach’s alpha and strong inter-correlation coefficients between items indicated high reliability and internal consistency of each of 5 domains in general, and in the 3 subdomains, which allows them to be used in further analyses.

Pharmacy students’ overall self-assessments of qualifications, competences, relevance, motivation and effectiveness in health promotion were significantly positive (Table 5). The surveyed students evaluated the relevance of health promotion the highest (overall evaluation 7.4 ± 1.8 on average), followed by their motivation for health promotion (7.1 ± 1.9 on average). The lowest assessments were reported for their own competences for health promotion (6.0 ± 1.9 on average). Between highest and lowest assessed domains were assessed qualifications and effectiveness of health promotion (6.5 ± 1.6 and 6.4 ± 1.8, respectively). Assessments of all domains were similar in 3 subscales covering them: health knowledge, disease prevention and coping with health problems.

**Figure 1:**
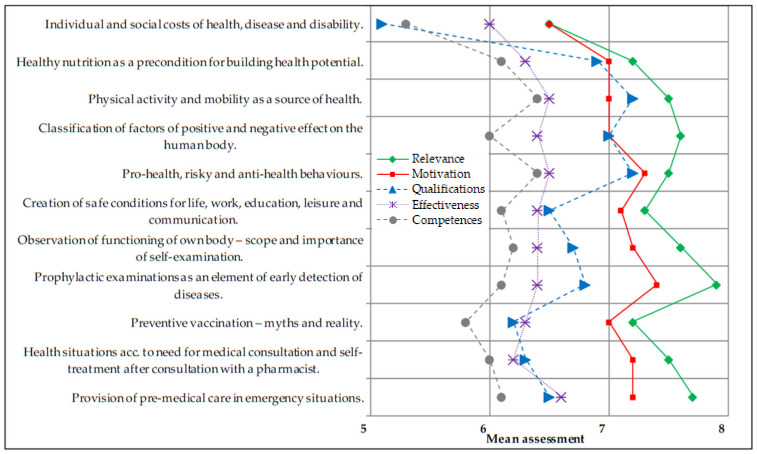
Pharmacy students’ mean assessment of qualifications, competences, relevance, motivation and effectiveness in health promotion—items in health knowledge (N = 206). Scale 1–10 where: 1—very low, 10—very high.

The surveyed pharmacy students’ assessments of qualifications, competences, relevance, motivation and effectiveness differed between the items in the subdomains. In the subdomain “health knowledge” (Figure 1), the lowest assessment was given to individual and social costs of health, diseases and disabilities, while the highest—to prophylactic examinations as an element of early detection of diseases.

**Figure 2:**
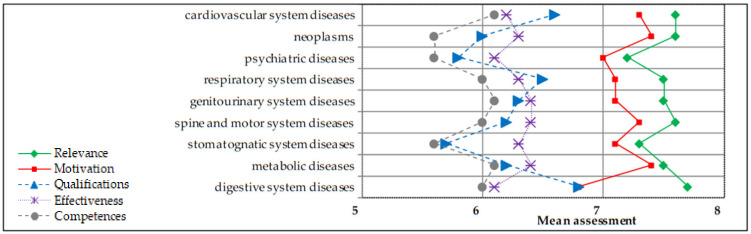
Pharmacy students’ mean assessment of qualifications, competences, relevance, motivation and effectiveness in health promotion—items in disease prevention (N = 206). Scale 1–10 where: 1—very low, 10—very high.

In the subdomain “disease prevention” (Figure 2), the lowest assessments were given to psychiatric and stomatognatic system disease prevention, while the highest to digestive system diseases prevention.

**Figure 3:**
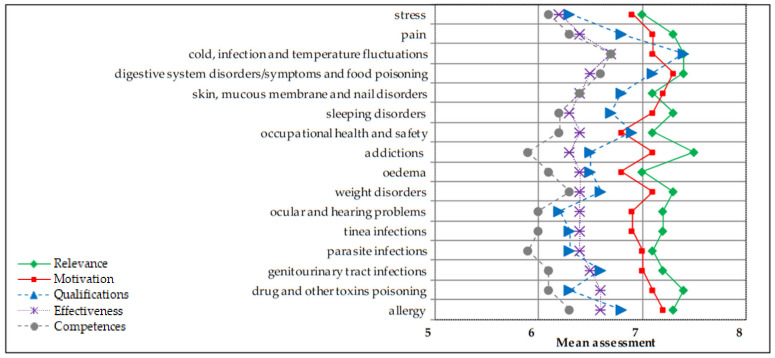
Pharmacy students’ mean assessment of qualifications, competences, relevance, motivation and effectiveness in health promotion—items in coping with health problems (N = 206). Scale 1–10 where: 1—very low, 10—very high.

In the subdomain “coping with health problems” (Figure 3), the highest assessment was given to coping with the cold, infection and temperature fluctuations.

## 4. Discussion

In the present study, the surveyed pharmacy students negatively assessed the pharmacists’ readiness to promote health in pharmacies from the Lublin region. In fact, they assessed the systemic solutions for health promotion as the worst and assessed the readiness of the professional group of pharmacists to such promotion as slightly better, however the assessments were negative. On the other hand, the surveyed students positively assessed their own readiness to promote health in pharmacies.

Pharmacy students in many countries, including Poland, see the need of health-related information provided to the patients [22,29,30,31]. Currently, a great commitment of students of Pharmacy Faculties all over Poland is observed. The students, under the supervision of specialized staff, take part in organizing pro-health campaigns that are very popular among the society. A survey conducted on a group of 209 pharmacy students showed that almost all of them considered a pharmacist as a person who should conduct health promotion when advising the patient [32]. In turn, the study performed on both pharmacy and medical students from Gdańsk (Poland) revealed that the medical students were not familiar with pharmaceutical care [22]. On the other hand, the pharmacy students pointed out that during their professional internship in pharmacies pharmaceutical care was implemented only to a small extent. In addition, according to the pharmacy students, pharmacists might be more engaged in the process of optimizing the pharmacotherapy [22]. 

Previously, the great potential of pharmacy students in health promotion was noticed and described [33]. Pharmacy students may, for example, conduct non-invasive screening tests for cardiovascular diseases (blood pressure measurement), or screening tests for Alzheimer’s disease. They may also educate patients in respect to both healthy diet and lifestyle. Anderson et al. [33] emphasized a need of including methodological strategies for health promotion and preventive healthcare used into the pharmaceutical studies program in the future.

Another survey for future pharmacists on their role in public health showed that they correctly understood the concept of “public health” [34]. Interestingly, the authors also pointed out that the students’ assessed their own knowledge on this subject as average. In addition, according to the students, the involvement of pharmacists in the protection of public health is not sufficient. On the other hand, in the United States the study by Rodis et al. [35] demonstrated that the education of pharmacy students in the field of health promotion during a 3-year education program was positively assessed. 

Worth noticing is also a high level of trust for pharmacists that Polish patients have—according to the survey performed by The Polish Pharmaceutical Chamber over 90% of patients trust their pharmacists. The introduction of pharmaceutical care to the pharmacies is supported by over 50% of patients, however nearly 40% of them don’t know what PC is. Most of the patients (over 60%) think that pharmacy is an important place of consultation on health matters, independent of their appointments with a physician [36]. Another study presented similar conclusion and showed that patients also expect pharmaceutical care as well as new educational services to be provided in pharmacies [37].

In our previous study on pharmacy staff working in community pharmacies [26] conducted using the same survey questionnaire, we obtained similar results to the current study on pharmacy students. The overall assessment of pharmacists’ readiness to promote health was negative—below the scale mid-point. The surveyed pharmacists rated the system solutions in the field of health promotion the lowest, and their readiness to promote health the highest. However, when comparing the assessments made by the pharmacy students in this study with the assessment made by the pharmacy staff in our previous study, it can be concluded that the surveyed pharmacy students rated general readiness, the system solutions, the readiness of the professional group of pharmacists and their own readiness to promote health significantly higher than the pharmacy staff. Both the pharmacy staff and the pharmacy students assessed the readiness to promote health better in a pharmacy than in the local community. A higher overall assessment of readiness for health promotion was reported by pharmacy staff who were women, younger people, having worked for no more than 5 years, with secondary education and being pharmacy managers.

In another study conducted by us previously using the same questionnaire but conducted among pharmacy staff working in community pharmacies [27], we obtained similar results to the current study on students. The surveyed pharmacy staff in the previous study, just as with the pharmacy students in this study, gave the highest scores to the relevance of health promotion, the lowest to their own competences and effectiveness in the field of health promotion in pharmacies. A higher self-assessment of qualifications, competences, relevance, motivation and effectiveness in the field of health promotion was experienced by pharmacy staff who were women, young people, with more than higher education, short work experience and being pharmacy managers. If we compare the assessments made by the pharmacy students in the current study with the assessment made by the pharmacy staff in our previous study, it can be concluded that the surveyed students assessed their qualifications, competences, relevance, motivation and effectiveness of health promotion in pharmacies significantly better than the surveyed pharmacy staff. The qualification, competences, relevance, motivation and effectiveness of health promotion in pharmacies were assessed significantly better by the surveyed pharmacy students than by the surveyed pharmacy staff also in these three areas: health knowledge, disease prevention and dealing with health problems.

The comparison of the results of the study performed among the pharmacy students and the results of the study performed among the pharmacists suggest that the students present more open and more optimistic attitudes on health promotion services provided in community pharmacies. That may be caused by a lack of experience working at a pharmacy and a knowledge of the working conditions.

Our study has some limitations. The first one is a lack of open questions in the questionnaire, which means that the students did not have a chance to freely present their opinions about health promotion. However, the goal of our study was to perform not qualitative, but quantitative research that allows for the generalization of the results.

Secondly, the participation in the survey was voluntary, so our results could be biased because the questionnaire was filled in only by those pharmacy students who were present at lectures and who filled it in correctly and completely. Another bias of social desirability could be as a typical difference between self-reported practices and actual practices [38,39].

Third, our study considered only supply aspects of health promotion and pharmaceutical care in pharmacies which in future will be provided by pharmacy students. Demand issues including expectations of different social groups, age groups or patients’ groups with various medical conditions and chronic diseases were not addressed but research in that area is planned by us in the nearest future.

## 5. Conclusions

Pharmacy students consider themselves ready to promote health, they consider it to be important and they are motivated to do so. They are a great potential, which can be used in order to improve the health care. It is important in the context of the new Act on the Profession of Pharmacist of 2020, which regulates the pharmaceutical care in Polish pharmacies. In growing number of countries, pharmaceutical care is also being improved and enhanced. One of the vital elements of pharmaceutical care is health promotion and health education. Medical universities, educating students and post-graduate students of pharmacy play a huge role in the proper development and functioning of pharmaceutical care. Legal, organizational and financial conditions should be created to encourage pharmacists to undertake individual education in the field of pharmaceutical care. Training course programs should cover issues not only related to pharmacotherapy, but also to epidemiology, health promotion, and especially communication with the patient in order to be able to take effective action to promote health. Pharmacy students represent a great potential that, in cooperation with other health care specialists, can be used in systematic and ad hoc activities related to health promotion and health prevention.

## Figures and Tables

**Table 1:** Pharmacy students’ readiness to promote health—overall score, systemic solutions, readiness as a professional group, personal readiness (N = 206).

Assessment of Readiness to Health Promotion	Cronbach’s Alpha	Average Inter-Correlation Coefficient	M ± SD	*p* ^1^
Overall score	0.948	0.370	4.9 ± 1.3	<0.001
Systemic solutions	0.940	0.507	4.0 ± 1.3	<0.001
Readiness as a professional group	0.909	0.537	5.0 ± 1.6	<0.001
Personal readiness	0.900	0.611	5.8 ± 1.7	0.032

^1^ One sample *t*-test against a test value of 5.5. M—mean, SD—standard deviation. Scale 1–10 where: 1—definitely no, 10—definitely yes.

**Table 2:** Pharmacy students’ assessment of systemic solutions for health promotion (N = 206).

Question	M ± SD	*p* ^1^
Is there a clearly defined (in your professional environment of pharmacists) concept of health promotion that includes prevention, local health policy and health education?	5.1 ± 1.9	0.001
Are there institutions or organizations that provide professional training for pharmacists in the area of health promotion, including health education?	4.7 ± 1.9	<0.001
Are there clear regulations that define and support a role of pharmacists in health promotion?	4.6 ± 1.9	<0.001
Is implementation of executive regulations and other guidelines for pharmacists in health promotion enforced by the pharmacy supervision body or professional associations?	4.3 ± 2.1	<0.001
Are there any institutions/organization that try in an orderly manner cooperate with pharmacists and support them in activities aimed at health promotion?	4.3 ± 1.8	<0.001
Do pharmacists have a working concept of coordinated activities aimed at assessing quality and effectiveness of health promotion activities performed by them?	4.1 ± 1.7	<0.001
Are there among pharmacists on a regional and local level people who may be considered spokespersons or leaders of the “Pharmacy promoting health” concept?	4.1 ± 1.9	<0.001
Is there any coordinated work among pharmacists on strategy for health promotion by pharmacists on a regional, local and institutional level?	4.0 ± 1.9	<0.001
Is there an effective system of support for pharmacists that delivers technical concepts for health promotion, technical support and information materials?	3.9 ± 1.7	<0.001
Does pharmacy professional association take actions aimed at improving competences of pharmacists as health promoters and at setting legal-organizational framework for such activities?	3.9 ± 1.8	<0.001
Do pharmacists have benchmark solutions for activities aimed at health promotion that can be used to work out their own health-educational programs?	3.8 ± 1.7	<0.001
Is it a common practice among pharmacists to use results of epidemiological and demographic research to plan activities in the area of health promotion and information?	3.6 ± 1.8	<0.001
Is there cooperation between local communities, local and central administration, and pharmacists focused on prophylactics/prevention of drug dependence and addiction, and on monitoring self-treatment?	3.6 ± 2.1	<0.001
Is there a system of financial support for pharmacies in performing health promotion activities, including health education, at workplace?	3.4 ± 1.8	<0.001
Is there a system of financial support for pharmacies in performing health promotion activities, including health education, in local communities?	3.4 ± 1.7	<0.001
Does pharmacy professional association take any action aimed at setting a financial framework for health promotion?	3.3 ± 1.7	<0.001

^1^ One sample *t*-test against a test value of 5.5. M—mean, SD—standard deviation. Scale 1–10 where: 1—definitely no, 10—definitely yes.

**Table 3:** Pharmacy students’ assessment of readiness to health promotion as a professional group (N = 206).

Question	At a Workplace		In a Local Community		Workplace vs. Local Community
	M ± SD	*p* ^1^	M ± SD	*p* ^1^	*p* ^2^
Are pharmacists ready to promote health and health education in terms of their knowledge?	5.8 ± 2.3	0.043	5.6 ± 2.3	0.372	0.008
Do pharmacists show positive motivation for active involvement in health promotion and health education?	5.5 ± 2.2	0.872	5.5 ± 2.1	0.769	0.808
Are pharmacists ready to promote health and health education in terms of methodology (objectives, forms, methods, influencing, methods of diagnosing patient/client, methods of evaluation and other)?	5.4 ± 2.1	0.718	5.3 ± 2.1	0.144	0.019
Do pharmacists initiate activities aimed at increasing their readiness to promote health?	4.9 ± 1.9	<0.001	4.8 ± 2.0	<0.001	0.300
Do pharmacists have appropriate conditions (premises, organizational, interpersonal) to promote health and health education?	3.9 ± 1.9	<0.001	3.7 ± 1.9	<0.001	<0.001

^1^ One sample *t*-test against a test value of 5.5. ^2^ Two paired samples *t* test. M—mean, SD—standard deviation. Scale 1–10 where: 1—definitely no, 10—definitely yes.

**Table 4:** Pharmacy students’ assessment of personal readiness to promote health (N = 206).

Question	M ± SD	*p* ^1^
Do you think you are ready to recognize and meet health expectations of patients/pharmacy clients to a larger extent than it is necessary for regular buy/sell relations?	6.2 ± 2.3	<0.001
Is level of your readiness to promote health at your workplace sufficient for effective actions?	5.9 ± 2.1	0.005
Is level of your readiness to promote health at your local community sufficient for effective actions?	5.9 ± 2.0	0.012
Do social attitude and personal relationships at workplace favor health promotion and health education?	5.7 ± 2.2	0.337
Do you think you are effective at health promotion?	5.7 ± 2.1	0.150
Do you have appropriate premises and organizational setting for health promotion activities?	5.2 ± 2.3	0.100

^1^ One sample *t*-test against a test value of 5.5. M—mean, SD—standard deviation. Scale 1–10 where: 1—definitely no, 10—definitely yes.

**Table 5:** Pharmacy students’ assessment of qualifications, competences, relevance, motivation and effectiveness in health promotion—domains and subdomains (N = 206).

Domain	Subdomain	Cronbach’s Alpha	Average Inter-Correlation Coefficient	M ± SD	*p* ^1^
Qualifications	Overall score	0.980	0.590	6.5 ± 1.6	<0.001
	Health knowledge	0.924	0.534	6.6 ± 1.5	<0.001
	Disease prevention	0.966	0.766	6.2 ± 1.9	<0.001
	Coping with health problems	0.974	0.713	6.6 ± 1.9	<0.001
Competences	Overall score	0.988	0.708	6.0 ± 1.9	<0.001
	Health knowledge	0.971	0.756	6.0 ± 1.9	<0.001
	Disease prevention	0.969	0.786	5.9 ± 2.0	0.004
	Coping with health problems	0.984	0.799	6.2 ± 2.1	<0.001
Relevance	Overall score	0.988	0.714	7.4 ± 1.8	<0.001
	Health knowledge	0.966	0.731	7.4 ± 1.9	<0.001
	Disease prevention	0.979	0.841	7.5 ± 1.9	<0.001
	Coping with health problems	0.984	0.799	7.2 ± 1.9	<0.001
Motivation	Overall score	0.992	0.775	7.1 ± 1.9	<0.001
	Health knowledge	0.974	0.782	7.1 ± 2.0	<0.001
	Disease prevention	0.977	0.832	7.2 ± 2.0	<0.001
	Coping with health problems	0.985	0.814	7.0 ± 2.0	<0.001
Effectiveness	Overall score	0.990	0.746	6.4 ± 1.8	<0.001
	Health knowledge	0.972	0.767	6.4 ± 1.8	<0.001
	Disease prevention	0.976	0.828	6.3 ± 1.9	<0.001
	Coping with health problems	0.983	0.763	6.4 ± 2.0	<0.001

^1^ One sample *t*-test against a test value of 5.5. M—mean, SD—standard deviation. Scale 1–10 where: 1—very low, 10—very high.

## Data Availability

The data presented in this study are available on request from the corresponding author. The data are not publicly available due to privacy restrictions.

## Supplementary Materials

The following are available online at https://www.mdpi.com/article/10.3390/ijerph182413227/s1. Supplementary Material S1—The questionnaire of readiness to promote health in pharmacies. Supplementary Material S2—The questionnaire of qualifications, competences, relevance, motivation and effectiveness of health promotion in pharmacies.
