# Supplementary material for: Polish Pharmacy Students’ Readiness, Qualifications, Competences, Relevance, Motivation and Effectiveness to Promote Health in Community Pharmacies
Source: Int J Environ Res Public Health. 2021 Dec 15;18(24):13227. doi: 10.3390/ijerph182413227 (PMC8701085; doi:10.3390/ijerph182413227)
Supplement: Supplementary file 1 [file ijerph-18-13227-s001.zip › supplementary 1.pdf]

The questionnaire of readiness to promote health in pharmacies

(based on the Scale of Social Readiness by Zbigniew Gaś)

Please put **X** to assess every item in a 10-point scale:

1 – definitely no, 10 – definitely yes.

1. Is there a clearly defined (in your professional environment of pharmacists) concept of health promotion that includes prevention, local health policy and health education?

1	2	3	4	5	6	7	8	9	10

2. Are there institutions or organizations that provide professional training for pharmacists in the area of health promotion, including health education?

1	2	3	4	5	6	7	8	9	10

3. Are there clear regulations that define and support a role of pharmacists in health promotion?

1	2	3	4	5	6	7	8	9	10

4. Is implementation of executive regulations and other guidelines for pharmacists in health promotion enforced by the pharmacy supervision body or professional associations?

1	2	3	4	5	6	7	8	9	10

5. Are there any institutions/organization that try in an orderly manner cooperate with pharmacists and support them in activities aimed at health promotion?

1	2	3	4	5	6	7	8	9	10

6. Do pharmacists have a working concept of coordinated activities aimed at assessing quality and effectiveness of health promotion activities performed by them?

1	2	3	4	5	6	7	8	9	10

7. Are there among pharmacists on a regional and local level people who may be considered spokespersons or leaders of the “Pharmacy promoting health” concept?

1	2	3	4	5	6	7	8	9	10

8. Is there any coordinated work among pharmacists on strategy for health promotion by pharmacists on a regional, local and institutional level?

1	2	3	4	5	6	7	8	9	10

9. Is there an effective system of support for pharmacists that delivers technical concepts for health promotion, technical support and information materials?

1	2	3	4	5	6	7	8	9	10

10. Does pharmacy professional association take actions aimed at improving competences of pharmacists as health promoters and at setting legal-organizational framework for such activities?

1	2	3	4	5	6	7	8	9	10

11. Do pharmacists have benchmark solutions for activities aimed at health promotion that can be used to work out their own health-educational programs?

1	2	3	4	5	6	7	8	9	10

12. Is it a common practice among pharmacists to use results of epidemiological and demographic research to plan activities in the area of health promotion and information?

1	2	3	4	5	6	7	8	9	10

13. Is there cooperation between local communities, local and central administration, and pharmacists focused on prophylactics/prevention of drug dependence and addiction, and on monitoring self-treatment?

1	2	3	4	5	6	7	8	9	10

14. Is there a system of financial support for pharmacies in performing health promotion activities, including health education, at workplace?

1	2	3	4	5	6	7	8	9	10

15. Is there a system of financial support for pharmacies in performing health promotion activities, including health education, in local communities?

1	2	3	4	5	6	7	8	9	10

16. Does pharmacy professional association take any action aimed at setting a financial framework for health promotion?

1	2	3	4	5	6	7	8	9	10

* * * * *

17. Are pharmacists ready to promote health and health education in terms of their knowledge <u>at a workplace</u>?

1	2	3	4	5	6	7	8	9	10

18. Are pharmacists ready to promote health and health education in terms of their knowledge <u>in a local community</u>?

1	2	3	4	5	6	7	8	9	10

19. Do pharmacists show positive motivation for active involvement in health promotion and health education <u>at a workplace</u>?

1	2	3	4	5	6	7	8	9	10

20. Do pharmacists show positive motivation for active involvement in health promotion and health education <u>in a local community</u>?

1	2	3	4	5	6	7	8	9	10

21. Are pharmacists ready to promote health and health education in terms of methodology (objectives, forms, methods, influencing, methods of diagnosing patient/client, methods of evaluation and other) <u>at a workplace</u>?

1	2	3	4	5	6	7	8	9	10

22. Are pharmacists ready to promote health and health education in terms of methodology (objectives, forms, methods, influencing, methods of diagnosing patient/client, methods of evaluation and other) in a local community?

1	2	3	4	5	6	7	8	9	10

23. Do pharmacists initiate activities aimed at increasing their readiness to promote health at a workplace?

1	2	3	4	5	6	7	8	9	10

24. Do pharmacists initiate activities aimed at increasing their readiness to promote health in a local community?

1	2	3	4	5	6	7	8	9	10

25. Do pharmacists have appropriate conditions (premises, organizational, interpersonal) to promote health and health education at a workplace?

1	2	3	4	5	6	7	8	9	10

26. Do pharmacists have appropriate conditions (premises, organizational, interpersonal) to promote health and health education in a local community?

1	2	3	4	5	6	7	8	9	10

* * * * *

27. Do you think you are ready to recognize and meet health expectations of patients/pharmacy clients to a larger extent than it is necessary for regular buy/sell relations?

1	2	3	4	5	6	7	8	9	10

28. Is level of your readiness to promote health at your workplace sufficient for effective actions?

1	2	3	4	5	6	7	8	9	10

29. Is level of your readiness to promote health at your local community sufficient for effective actions?

1	2	3	4	5	6	7	8	9	10

30. Do social attitude and personal relationships at workplace favor health promotion and health education?

1	2	3	4	5	6	7	8	9	10

31. Do you think you are effective at health promotion?

1	2	3	4	5	6	7	8	9	10

32. Do you have appropriate premises and organizational setting for health promotion activities?

1	2	3	4	5	6	7	8	9	10

*****

Gender: □ woman □ man
