# Supplementary material for: Polish Pharmacy Students’ Readiness, Qualifications, Competences, Relevance, Motivation and Effectiveness to Promote Health in Community Pharmacies
Source: Int J Environ Res Public Health. 2021 Dec 15;18(24):13227. doi: 10.3390/ijerph182413227 (PMC8701085; doi:10.3390/ijerph182413227)
Supplement: Supplementary file 1 [file ijerph-18-13227-s001.zip › supplementary 2.pdf]

**The questionnaire**
**of qualifications, competences, relevance, motivation and effectiveness**
**of health promotion in pharmacies**

Please put **X** to assess every item in a 10-point scale:
1 – very low, 10 – very high.

*************************************************************

## I. Qualifications

How do you assess your qualifications to promote health in a pharmacy in the following items?

| | **Health knowledge** | **1** | **2** | **3** | **4** | **5** | **6** | **7** | **8** | **9** | **10** |
|---|---|---|---|---|---|---|---|---|---|---|---|
| 1. | Individual and social costs of health, disease and disability | | | | | | | | | | |
| 2. | Healthy nutrition as a precondition for building health potential | | | | | | | | | | |
| 3. | Physical activity and mobility as a source of health | | | | | | | | | | |
| 4. | Classification of factors of positive and negative effect on the human body | | | | | | | | | | |
| 5. | Pro-health, risky and anti-health behaviours | | | | | | | | | | |
| 6. | Creation of safe conditions for life, work, education, leisure and communication | | | | | | | | | | |
| 7. | Observation of functioning of own body – scope and importance of self-examination | | | | | | | | | | |
| 8. | Prophylactic examinations as an element of early detection of diseases | | | | | | | | | | |
| 9. | Preventive vaccination – myths and reality | | | | | | | | | | |
| 10. | Health situations acc. to need for medical consultation and self-treatment after consultation with a pharmacist | | | | | | | | | | |
| 11. | Provision of pre-medical care in emergency situations | | | | | | | | | | |

| | | | | | | | | | | | |
|---|---|---|---|---|---|---|---|---|---|---|---|
| | **Prevention of** | **1** | **2** | **3** | **4** | **5** | **6** | **7** | **8** | **9** | **10** |
| 12. | cardiovascular system diseases | | | | | | | | | | |
| 13. | neoplasms | | | | | | | | | | |
| 14. | psychiatric diseases | | | | | | | | | | |
| 15. | respiratory system diseases | | | | | | | | | | |
| 16. | genitourinary system diseases | | | | | | | | | | |
| 17. | spine and motor system diseases | | | | | | | | | | |
| 18. | stomatognatic system diseases | | | | | | | | | | |
| 19. | metabolic diseases | | | | | | | | | | |
| 20. | digestive system diseases | | | | | | | | | | |
| | | | | | | | | | | | |

| | **Copying with** | **1** | **2** | **3** | **4** | **5** | **6** | **7** | **8** | **9** | **10** |
|---|---|---|---|---|---|---|---|---|---|---|---|
| 21. | stress | | | | | | | | | | |
| 22. | pain | | | | | | | | | | |
| 23. | cold, infection and temperature fluctuations | | | | | | | | | | |
| 24. | digestive system disorders/symptoms and food poisoning | | | | | | | | | | |
| 25. | skin, mucous membrane and nail disorders | | | | | | | | | | |
| 26. | sleeping disorders | | | | | | | | | | |
| 27. | occupational health and safety | | | | | | | | | | |
| 28. | addictions | | | | | | | | | | |
| 29. | oedema | | | | | | | | | | |
| 30. | weight disorders | | | | | | | | | | |
| 31. | ocular and hearing problems | | | | | | | | | | |
| 32. | tinea infections | | | | | | | | | | |
| 33. | parasite infections | | | | | | | | | | |
| 34 | genitourinary tract infections | | | | | | | | | | |
| 35. | drug and other toxins poisoning | | | | | | | | | | |
| 36. | allergy | | | | | | | | | | |

## II. Competences

How do you assess your formal competences to promote health in a pharmacy in the following items?

| | **Health knowledge** | **1** | **2** | **3** | **4** | **5** | **6** | **7** | **8** | **9** | **10** |
|---|---|---|---|---|---|---|---|---|---|---|---|
| 1. | Individual and social costs of health, disease and disability | | | | | | | | | | |
| 2. | Healthy nutrition as a precondition for building health potential | | | | | | | | | | |
| 3. | Physical activity and mobility as a source of health | | | | | | | | | | |
| 4. | Classification of factors of positive and negative effect on the human body | | | | | | | | | | |
| 5. | Pro-health, risky and anti-health behaviours | | | | | | | | | | |
| 6. | Creation of safe conditions for life, work, education, leisure and communication | | | | | | | | | | |
| 7. | Observation of functioning of own body – scope and importance of self-examination | | | | | | | | | | |
| 8. | Prophylactic examinations as an element of early detection of diseases | | | | | | | | | | |
| 9. | Preventive vaccination – myths and reality | | | | | | | | | | |
| 10. | Health situations acc. to need for medical consultation and self-treatment after consultation with a pharmacist | | | | | | | | | | |
| 11. | Provision of pre-medical care in emergency situations | | | | | | | | | | |

| | | | | | | | | | | | |
|---|---|---|---|---|---|---|---|---|---|---|---|
| | **Prevention of** | **1** | **2** | **3** | **4** | **5** | **6** | **7** | **8** | **9** | **10** |
| 12. | cardiovascular system diseases | | | | | | | | | | |
| 13. | neoplasms | | | | | | | | | | |
| 14. | psychiatric diseases | | | | | | | | | | |
| 15. | respiratory system diseases | | | | | | | | | | |
| 16. | genitourinary system diseases | | | | | | | | | | |
| 17. | spine and motor system diseases | | | | | | | | | | |
| 18. | stomatognatic system diseases | | | | | | | | | | |
| 19. | metabolic diseases | | | | | | | | | | |
| 20. | digestive system diseases | | | | | | | | | | |
| | | | | | | | | | | | |

| | **Copying with** | **1** | **2** | **3** | **4** | **5** | **6** | **7** | **8** | **9** | **10** |
|---|---|---|---|---|---|---|---|---|---|---|---|
| 21. | stress | | | | | | | | | | |
| 22. | pain | | | | | | | | | | |
| 23. | cold, infection and temperature fluctuations | | | | | | | | | | |
| 24. | digestive system disorders/symptoms and food poisoning | | | | | | | | | | |
| 25. | skin, mucous membrane and nail disorders | | | | | | | | | | |
| 26. | sleeping disorders | | | | | | | | | | |
| 27. | occupational health and safety | | | | | | | | | | |
| 28. | addictions | | | | | | | | | | |
| 29. | oedema | | | | | | | | | | |
| 30. | weight disorders | | | | | | | | | | |
| 31. | ocular and hearing problems | | | | | | | | | | |
| 32. | tinea infections | | | | | | | | | | |
| 33. | parasite infections | | | | | | | | | | |
| 34 | genitourinary tract infections | | | | | | | | | | |
| 35. | drug and other toxins poisoning | | | | | | | | | | |
| 36. | allergy | | | | | | | | | | |

**III. Relevance**

How do you assess relevance of health promotion in a pharmacy in the following items?

| | Health knowledge | 1 | 2 | 3 | 4 | 5 | 6 | 7 | 8 | 9 | 10 |
|---|---|---|---|---|---|---|---|---|---|---|---|
| 1. | Individual and social costs of health, disease and disability | | | | | | | | | | |
| 2. | Healthy nutrition as a precondition for building health potential | | | | | | | | | | |
| 3. | Physical activity and mobility as a source of health | | | | | | | | | | |
| 4. | Classification of factors of positive and negative effect on the human body | | | | | | | | | | |
| 5. | Pro-health, risky and anti-health behaviours | | | | | | | | | | |
| 6. | Creation of safe conditions for life, work, education, leisure and communication | | | | | | | | | | |
| 7. | Observation of functioning of own body – scope and importance of self-examination | | | | | | | | | | |
| 8. | Prophylactic examinations as an element of early detection of diseases | | | | | | | | | | |
| 9. | Preventive vaccination – myths and reality | | | | | | | | | | |
| 10. | Health situations acc. to need for medical consultation and self-treatment after consultation with a pharmacist | | | | | | | | | | |
| 11. | Provision of pre-medical care in emergency situations | | | | | | | | | | |

| | | | | | | | | | | | |
|---|---|---|---|---|---|---|---|---|---|---|---|
| | | | | | | | | | | | |
| | **Prevention of** | **1** | **2** | **3** | **4** | **5** | **6** | **7** | **8** | **9** | **10** |
| 12. | cardiovascular system diseases | | | | | | | | | | |
| 13. | neoplasms | | | | | | | | | | |
| 14. | psychiatric diseases | | | | | | | | | | |
| 15. | respiratory system diseases | | | | | | | | | | |
| 16. | genitourinary system diseases | | | | | | | | | | |
| 17. | spine and motor system diseases | | | | | | | | | | |
| 18. | stomatognatic system diseases | | | | | | | | | | |
| 19. | metabolic diseases | | | | | | | | | | |
| 20. | digestive system diseases | | | | | | | | | | |
| | | | | | | | | | | | |

| | **Copying with** | **1** | **2** | **3** | **4** | **5** | **6** | **7** | **8** | **9** | **10** |
|---|---|---|---|---|---|---|---|---|---|---|---|
| 21. | stress | | | | | | | | | | |
| 22. | pain | | | | | | | | | | |
| 23. | cold, infection and temperature fluctuations | | | | | | | | | | |
| 24. | digestive system disorders/symptoms and food poisoning | | | | | | | | | | |
| 25. | skin, mucous membrane and nail disorders | | | | | | | | | | |
| 26. | sleeping disorders | | | | | | | | | | |
| 27. | occupational health and safety | | | | | | | | | | |
| 28. | addictions | | | | | | | | | | |
| 29. | oedema | | | | | | | | | | |
| 30. | weight disorders | | | | | | | | | | |
| 31. | ocular and hearing problems | | | | | | | | | | |
| 32. | tinea infections | | | | | | | | | | |
| 33. | parasite infections | | | | | | | | | | |
| 34 | genitourinary tract infections | | | | | | | | | | |
| 35. | drug and other toxins poisoning | | | | | | | | | | |
| 36. | allergy | | | | | | | | | | |

## IV. Motivations

How do you assess your motivations to promote health in a pharmacy in the following items?

| | **Health knowledge** | **1** | **2** | **3** | **4** | **5** | **6** | **7** | **8** | **9** | **10** |
|---|---|---|---|---|---|---|---|---|---|---|---|
| 1. | Individual and social costs of health, disease and disability | | | | | | | | | | |
| 2. | Healthy nutrition as a precondition for building health potential | | | | | | | | | | |
| 3. | Physical activity and mobility as a source of health | | | | | | | | | | |
| 4. | Classification of factors of positive and negative effect on the human body | | | | | | | | | | |
| 5. | Pro-health, risky and anti-health behaviours | | | | | | | | | | |
| 6. | Creation of safe conditions for life, work, education, leisure and communication | | | | | | | | | | |
| 7. | Observation of functioning of own body – scope and importance of self-examination | | | | | | | | | | |
| 8. | Prophylactic examinations as an element of early detection of diseases | | | | | | | | | | |
| 9. | Preventive vaccination – myths and reality | | | | | | | | | | |
| 10. | Health situations acc. to need for medical consultation and self-treatment after consultation with a pharmacist | | | | | | | | | | |
| 11. | Provision of pre-medical care in emergency situations | | | | | | | | | | |

| | | | | | | | | | | | |
|---|---|---|---|---|---|---|---|---|---|---|---|
| | **Prevention of** | **1** | **2** | **3** | **4** | **5** | **6** | **7** | **8** | **9** | **10** |
| 12. | cardiovascular system diseases | | | | | | | | | | |
| 13. | neoplasms | | | | | | | | | | |
| 14. | psychiatric diseases | | | | | | | | | | |
| 15. | respiratory system diseases | | | | | | | | | | |
| 16. | genitourinary system diseases | | | | | | | | | | |
| 17. | spine and motor system diseases | | | | | | | | | | |
| 18. | stomatognatic system diseases | | | | | | | | | | |
| 19. | metabolic diseases | | | | | | | | | | |
| 20. | digestive system diseases | | | | | | | | | | |
| | | | | | | | | | | | |

| | **Copying with** | **1** | **2** | **3** | **4** | **5** | **6** | **7** | **8** | **9** | **10** |
|---|---|---|---|---|---|---|---|---|---|---|---|
| 21. | stress | | | | | | | | | | |
| 22. | pain | | | | | | | | | | |
| 23. | cold, infection and temperature fluctuations | | | | | | | | | | |
| 24. | digestive system disorders/symptoms and food poisoning | | | | | | | | | | |
| 25. | skin, mucous membrane and nail disorders | | | | | | | | | | |
| 26. | sleeping disorders | | | | | | | | | | |
| 27. | occupational health and safety | | | | | | | | | | |
| 28. | addictions | | | | | | | | | | |
| 29. | oedema | | | | | | | | | | |
| 30. | weight disorders | | | | | | | | | | |
| 31. | ocular and hearing problems | | | | | | | | | | |
| 32. | tinea infections | | | | | | | | | | |
| 33. | parasite infections | | | | | | | | | | |
| 34 | genitourinary tract infections | | | | | | | | | | |
| 35. | drug and other toxins poisoning | | | | | | | | | | |
| 36. | allergy | | | | | | | | | | |

V. **Effectiveness**

How do you assess your effectiveness in health promotion in a pharmacy in the following items?

| | **Health knowledge** | **1** | **2** | **3** | **4** | **5** | **6** | **7** | **8** | **9** | **10** |
|---|---|---|---|---|---|---|---|---|---|---|---|
| 1. | Individual and social costs of health, disease and disability | | | | | | | | | | |
| 2. | Healthy nutrition as a precondition for building health potential | | | | | | | | | | |
| 3. | Physical activity and mobility as a source of health | | | | | | | | | | |
| 4. | Classification of factors of positive and negative effect on the human body | | | | | | | | | | |
| 5. | Pro-health, risky and anti-health behaviours | | | | | | | | | | |
| 6. | Creation of safe conditions for life, work, education, leisure and communication | | | | | | | | | | |
| 7. | Observation of functioning of own body – scope and importance of self-examination | | | | | | | | | | |
| 8. | Prophylactic examinations as an element of early detection of diseases | | | | | | | | | | |
| 9. | Preventive vaccination – myths and reality | | | | | | | | | | |
| 10. | Health situations acc. to need for medical consultation and self-treatment after consultation with a pharmacist | | | | | | | | | | |
| 11. | Provision of pre-medical care in emergency situations | | | | | | | | | | |

| | | | | | | | | | | | |
|---|---|---|---|---|---|---|---|---|---|---|---|
| | **Prevention of** | **1** | **2** | **3** | **4** | **5** | **6** | **7** | **8** | **9** | **10** |
| 12. | cardiovascular system diseases | | | | | | | | | | |
| 13. | neoplasms | | | | | | | | | | |
| 14. | psychiatric diseases | | | | | | | | | | |
| 15. | respiratory system diseases | | | | | | | | | | |
| 16. | genitourinary system diseases | | | | | | | | | | |
| 17. | spine and motor system diseases | | | | | | | | | | |
| 18. | stomatognatic system diseases | | | | | | | | | | |
| 19. | metabolic diseases | | | | | | | | | | |
| 20. | digestive system diseases | | | | | | | | | | |
| | | | | | | | | | | | |

| | **Copying with** | **1** | **2** | **3** | **4** | **5** | **6** | **7** | **8** | **9** | **10** |
|---|---|---|---|---|---|---|---|---|---|---|---|
| 21. | stress | | | | | | | | | | |
| 22. | pain | | | | | | | | | | |
| 23. | cold, infection and temperature fluctuations | | | | | | | | | | |
| 24. | digestive system disorders/symptoms and food poisoning | | | | | | | | | | |
| 25. | skin, mucous membrane and nail disorders | | | | | | | | | | |
| 26. | sleeping disorders | | | | | | | | | | |
| 27. | occupational health and safety | | | | | | | | | | |
| 28. | addictions | | | | | | | | | | |
| 29. | oedema | | | | | | | | | | |
| 30. | weight disorders | | | | | | | | | | |
| 31. | ocular and hearing problems | | | | | | | | | | |
| 32. | tinea infections | | | | | | | | | | |
| 33. | parasite infections | | | | | | | | | | |
| 34 | genitourinary tract infections | | | | | | | | | | |
| 35. | drug and other toxins poisoning | | | | | | | | | | |
| 36. | allergy | | | | | | | | | | |
